# Supplementary material for: Replacing Sedentary Time with Physical Activity and Sleep: A 24-Hour Movement Behaviour Perspective on Appetite Control
Source: Nutrients. 2025 Oct 7;17(19):3163. doi: 10.3390/nu17193163 (PMC12526340; doi:10.3390/nu17193163)

Supplementary Table S1. Participant characteristics relating to appetite perceptions, appetite-related hormone and food reward outcomes (n = 119).

**Daily energy intake**	
Laboratory measured	1973 ± 742 kcal
Free-living (food diary)	2467 ± 706 kcal
Fasting leptin (pg·mL^{-1})	6312 (12272)
Fasting PYY (pg·mL^{-1})	127 ± 52
PYY AUC (2 h, pg·mL^{-1})	19880 ± 6669
Fasting acylated ghrelin (pg·mL^{-1})	133 (145)
Acylated ghrelin AUC (2 h, pg·mL^{-1})	9879 (11506)
**Perceived ratings of appetite**	
Fasting fullness (mm)	25 ± 21
Fullness AUC (4 h, mm)	11247 ± 4368
Fasting hunger (mm)	53 ± 25
Hunger AUC (4 h, mm)	10922 ± 5150
Fasting PFC (mm)	63 ± 19
PFC AUC (4 h, mm)	12742 ± 4878
Fasting satisfaction (mm)	52 ± 23
Satisfaction AUC (4 h, mm)	14148 ± 4210
**TFEQ**	
Cognitive restraint (0-21)	6.5 ± 4.1
Disinhibition (0-16)	5.2 ± 2.8
Hunger (0-14)	5.5 ± 3.2
**CoEQ**	
Craving control	287.9 ± 67.3
Craving for Sweet	157.2 ± 84.4
Craving for Savoury	185.8 ± 77.4
Positive Mood	249.8 ± 67.2
**LFPQ**	
Fasting fat explicit liking (mm)	5.0 ± 13.1
Fasting fat explicit wanting (mm)	4.5 ± 13.3
Fasting fat implicit wanting (AU)	13.1 ± 26.9
Fasting fat relative preference (AU)	5.0 ± 9.8
Fasting taste explicit liking (mm)	-1.0 ± 17.6
Fasting taste explicit wanting (mm)	-0.6 ± 17.2
Fasting taste implicit wanting (AU)	-2.6 ± 36.9
Fasting taste relative preference (AU)	-0.8 ± 13.4

Data are presented for *n* = 119. Values are mean ± standard deviation (SD) for normally distributed variables, median and interquartile range (IQR) for non-normally distributed variables, and frequencies and percentages for categorical variables. AU, arbitrary units; AUC, area under the curve; CoEQ, Control of Eating Questionnaire; LFPQ, Leeds Food Preference Questionnaire; PFC, prospective food consumption; PYY, peptide-YY; TFEQ, Three-Factor Eating Questionnaire.

Supplementary Table S2. Adjusted change in perceived ratings of appetite and appetite-related hormones associated with replacing 30 min of sedentary time with sleep or physical activity in Model 1 (adjusted for age, sex, ethnicity, and smoking status).

	LPA		MVPA		Sleep	
	β-coefficient (95% CI)	**P**-value	β -coefficient (95% CI)	**P**-value	β -coefficient (95% CI)	**P**-value
Appetite-related hormones						
Fasting leptin (pg·mL^{-1})*	0.06 (−0.03, 0.15)	0.064	−0.08 (−0.17, 0.01)	0.063	**−0.08 (−0.14, −0.01)**	0.029
Fasting PYY (pg·mL^{-1})	5.0 (−1.4, 11.4)	0.123	−0.9 (−7.2, 5.5)	0.787	−0.4 (−4.7, 4.0)	0.864
PYY AUC (2 h, pg·mL^{-1})	472.0 (−43.9, 987.7)	0.073	**−616.3 (−1126.9, −105.7)**	**0.018**	82.2 (−268.5, 432.9)	0.646
Fasting acylated ghrelin (pg·mL^{-1})*	0.04 (−0.06, 0.14)	0.589	0.09 (0.00, 0.20)	0.055	0.00 (−0.06, 0.06)	0.963
Acylated Ghrelin AUC (2 h, pg·mL^{-1})*	−0.03 (−0.06, 0.03)	0.298	−0.03 (−0.06, 0.03)	0.543	0.00 (−0.06, 0.03)	0.461
Perceived ratings of appetite						
Fasting fullness (mm)	−0.15 (−2.79, 2.48)	0.913	0.97 (−1.68, 3.62)	0.481	0.04 (−1.85, 1.90)	0.984
Fullness AUC (4 h, mm)	54.7 (−429.7, 539.0)	0.825	−468.9 (−953.9, 16.2)	0.058	−304.1 (−636.7, 28.5)	0.073
Fasting hunger (mm)	−0.4 (−3.5, 2.8)	0.816	0.8 (−2.3, 4.0)	0.610	0.1 (−2.1, 2.3)	0.938
Hunger AUC (4 h, mm)	−126.2 (−658.6, 406.2)	0.642	495.9 (−36.6, 1028.5)	0.068	319.5 (−45.9m 684.9)	0.225
Fasting PFC (mm)	0.34 (−1.77, 2.46)	0.750	1.78 (−0.37, 3.91)	0.103	0.50 (−0.93, 1.99)	0.480
PFC AUC (4 h, mm)	−197.3 (−644.3m 249.7)	0.387	**553.4 (101.9, 1004.9)**	**0.016**	186.3 (−121.1, 493.7)	0.235
Fasting satisfaction (mm)	1.44 (−1.41, 4.32)	0.322	0.51 (−2.37, 3.39)	0.731	0.88 (−1.11, 2.83)	0.390
Satisfaction AUC (4 h, mm)	−142.3 (−620.5, 335.8)	0.560	−134.3 (−610.6, 342.0)	0.580	−191.5 (−519.5, 136.6)	0.253

Each coefficient reflects the predicted change in the specified appetite-related outcome associated with reallocating 30 minutes of sedentary time to the specified behavior (LPA, MVPA, sleep), holding total time constant. Values are presented as adjusted means with 95% CI for *n* = 119. Data were analysed using generalised linear models with a normal distribution and identity link function or gamma distribution with a log link function indicated by *. Model 1 adjusted for age, sex, smoking status, and ethnicity. AU, arbitrary units; CI, confidence interval; LPA, light physical activity; MVPA, moderate-to-vigorous physical activity. * Values represent coefficients on the log-scale for models with gamma distribution and log link function.

Supplementary Table S3. Adjusted change in food reward (LFPQ), food cravings (CoEQ) and eating traits (TFEQ) associated with replacing 30 min of sedentary time with sleep or physical activity in Model 1 (adjusted for age, sex, ethnicity, and smoking status).

	LPA		MVPA		Sleep	
	β -coefficient (95% CI)	**P**-value	β -coefficient (95% CI)	**P**-value	β -coefficient (95% CI)	**P**-value
TFEQ						
Cognitive restraint (0-21)	0.12 (−0.39, 0.63)	0.665	−0.06 (−0.57, 0.45)	0.817	−0.15 (−0.47, 0.21)	0.406
Disinhibition (0-16)	0.00 (−0.33, 0.33)	0.973	0.10 (−0.29, 0.48)	0.633	−0.11 (−0.33, 0.11)	0.276
Hunger (0-14)	0.19 (−0.21, 0.57)	0.358	0.31 (−0.09, 0.70)	0.120	−0.03 (−0.27, 0.25)	0.878
Total score	0.27 (−0.50, 1.02)	0.503	0.34 (−0.43, 1.12)	0.393	−0.30 (−0.84, 0.24)	0.241
CoEQ						
Craving control (AU)	−5.55 (−13.68, 2.58)	0.181	2.73 (−5.40, 10.86)	0.510	3.88 (−1.71, 9.48)	0.176
Craving for Sweet (AU)	2.36 (−7.55, 12.27)	0.641	−2.90 (−12.80, 6.99)	0.564	6.07 (−0.66, 12.81)	0.078
Craving for Savoury (AU)	3.3 (−6.1, 12.7)	0.490	2.0 (−7.4, 11.3)	0.677	−3.7 (−10.1, 2.7)	0.253
Positive Mood (AU)	5.1 (−17.9, 28.2)	0.661	−6.0 (−22.8, 10.8)	0.127	3.9 (−14.4, 22.3)	0.152
LFPQ						
Fasting fat explicit liking (mm)	−0.3 (−1.6, 1.3)	0.724	0.0 (−1.6, 1.6)	0.973	−0.4 (−1.4, 0.7)	0.519
Fasting fat explicit wanting (mm)	−0.1 (−1.6, 1.5)	0.882	0.5 (−1.0, 2.0)	0.502	−0.1 (−1.1, 1.0)	0.920
Fasting fat implicit wanting (AU)	−0.6 (−3.8, 2.6)	0.726	−0.3 (−3.2, 2.7)	0.869	−0.5 (−2.6, 1.7)	0.682
Fasting fat relative preference (AU)	−0.21 (−1.38, 0.96)	0.719	−0.21 (−1.38, 0.96)	0.722	0.12 (−0.69, 0.93)	0.762
Fasting taste explicit liking (mm)	−0.78 (−3.00, 1.43)	0.486	−0.81 (−3.03, 1.41)	0.478	−0.09 (−1.59, 1.41)	0.920
Fasting taste explicit wanting (mm)	−0.78 (−2.94, 1.38)	0.486	−0.39 (−2.56, 1.78)	0.728	0.36 (−1.14, 1.86)	0.635

Fasting taste implicit wanting (AU)	0.78 (−3.81, 5.37)	0.743	−3.57 (−8.18, 1.04)	0.128	0.99 (−2.16, 4.14)	0.538
Fasting taste relative preference (AU)	0.00 (−5.56, 5.56)	0.996	−2.30 (−7.90, 3.30)	0.420	0.50 (−3.30, 4.30)	0.799

Each coefficient reflects the predicted change in the specified appetite-related outcome associated with reallocating 30 minutes of sedentary time to the specified behavior (LPA, MVPA, sleep), holding total time constant. Values are presented as adjusted means with 95% CI for *n* = 119. Data were analysed using generalised linear models with a normal distribution and identity link function. Model 1 adjusted for age, sex, smoking status, and ethnicity. AU, arbitrary units; CI, confidence interval; CoEQ, Control of Eating Questionnaire; ES, effect size; LFPQ, Leeds Food Preference Questionnaire; LPA, light physical activity; MVPA, moderate-to-vigorous physical activity; TFEQ, Three-Factor Eating Questionnaire.

Supplementary Figure S1 - Adjusted change in energy intake (measured in the laboratory and field) associated with replacing 30 min of sedentary time with sleep or physical activity in Model 1 (adjusted for age, sex, ethnicity and smoking status).

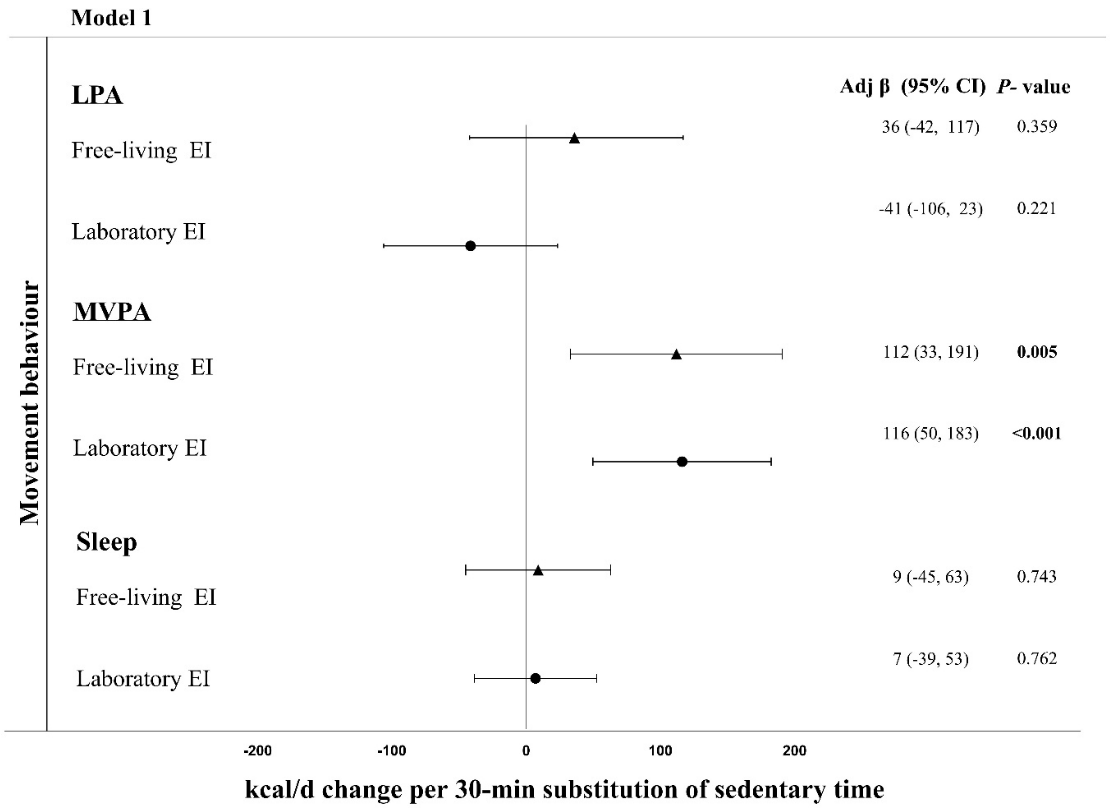

Supplement: Supplementary file 1 [file nutrients-17-03163-s001.zip › nutrients-3891199-supplementary.pdf]
